# *In silico* drug repurposing using molecular docking and dynamics to target the protein interaction between the SARS-CoV-2 S-glycoprotein and the ACE2 receptor

**DOI:** 10.12688/f1000research.131508.1

**Published:** 2023-11-09

**Authors:** Dania Hussein, Abdullah Almatrafi, Mohammed Gomaa, AlAnood Alhowsawi, Sarah Almustafa, Hadi Alsaihaty, Manar Alghamdi

**Affiliations:** 1 Pharmacology and Toxicology, Imam Abdulrahman bin Faisal University, Khobar, Saudi Arabia; 2 Pharmaceutical Chemistry, Imam Abdulrahman bin Faisal University, Khobar, Saudi Arabia; 3 Imam Abdulrahman bin Faisal University, Khobar, Saudi Arabia

**Keywords:** protein interaction, Sars-CoV-2, S-glycoprotein, Angiotensin converting enzyme-2, molecular dynamics, drug repurposing.

## Abstract

**Background:** The protein interaction between the viral surface S-glycoprotein and the host angiotensin converting enzyme-2 receptor (ACE2) is key to the virulent nature of SARS-CoV-2. The potential role that effective drug repurposing strategies may have to help stem the impact of future outbreaks has been brought to light in the recent COVID-19 pandemic. This study outlines a comprehensive approach towards *in-silico* drug discovery which aims to identify hit agents that can be suitably translated into a clinical setting.

**Methods:** We use two different computational platforms to analyze the viral S-glycoprotein in its bound conformational state to the ACE2 receptor. We employed a comprehensive screening approach to shortlist compounds capable of binding to the viral target interface and corroborated these findings using both Schrödinger’s Glide and AutoDock Vina. Molecular dynamic simulation studies further verified the stability of the interaction at the viral-host protein interface.

**Results:** Lymecycline, pentagalloylglucose, polydatin, and hexoprenaline were identified as prime candidates for further studies given the robust and stable nature of their interaction at the viral-host interface and relevance for clinical testing. These agents were shown in a 100-nanosecond simulation trajectory to favorably disrupt key binding interactions at the viral-host interface and may potentially inhibit viral entry into host cells. In all hit molecules it was observed that inhibiting the interaction with the following key viral binding residues: Lys17, Gly496, Tyr 505, and key host residues: His34, Asp38, Lys353, played a critical role toward the inhibition of the viral-host protein interaction.

**Conclusions:** Our study is unique in its comprehensive approach to identify agents that can bind to the S-glycoprotein-ACE2 interface using multiple computational platforms. Among the hit compounds shortlisted in this study, both lymecycline and hexoprenaline may be considered as candidates for preliminarily clinical studies to assess their therapeutic potential in the management of COVID-19 infections.

## Introduction

The novel corona virus, severe acute respiratory syndrome coronavirus 2 (SARS-CoV-2), has had an unprecedented impact worldwide. It is the viral entity responsible for the Coronavirus disease 2019 (COVID-19) pandemic, which has reaped significant detrimental effects upon societies and economies worldwide. Up until present day, efforts remain ongoing towards effective infection management and control. ^1^

The SARS-CoV-2 virus is an enveloped single stranded RNA virus. ^2^ The entire viral genome has been sequenced and found to comprise of 29,881 bp encoding 9860 amino acids (GenBank no. MN908947). ^2^ The viral RNA codes for both structural and non-structural proteins, most notable of which are the structural S-glycoproteins or ‘spike proteins’- key to viral-host attachment and infection. ^2^ The S-glycoproteins are responsible for binding to the angiotensin converting enzyme 2 (ACE2) host cell receptor and initiating viral entry into the host cell. ^3^ Both SARS-CoV and SARS-CoV-2 bind to the host ACE2 receptor to trigger viral entry and infection, however the binding affinity of the SARS-CoV-2 S-glycoprotein to ACE2 is shown to be over 20 times greater than that of SARS-CoV S-glycoprotein and ACE2 binding interaction. ^2^^,^^4^

The viral S-glycoprotein is a trimeric protein with a characteristic ‘stalk and halo’ like appearance. ^2^ Its peptide chain, composed of a total of 1237 amino acids, includes the S1 (aa residues 14–685) and S2 subunits (aa residues 686–1273), which have been characterized and found to be critical for viral attachment and membrane fusion. ^5^^,^^6^ In these regions, both the critical receptor binding domain (aa residues 319–541), as well as the fusion peptide domain (aa residues 788–806), were identified and characterized. ^4^^,^^7^

While vaccination efforts have greatly stemmed the spread of the infection, the main drawback of long-lasting vaccine efficacy appears to be the growing incidence of variants. ^8^ Managing the disease with currently available and approved therapeutics, remains an essential approach for treatment. However, there appears to be is no consensus on effective management strategies yet. ^9^^,^^10^ Drug repurposing is an approach to identify novel indications and uses for approved medications. In the era of COVID-19 drug repurposing has been shown to be an essential path towards identifying potentially effective therapies for disease management. ^11^^,^^12^ Strategies towards this include *in silico* drug discovery tools and virtual screening, which have proven to be most valuable in identifying potential drugs that can be repurposed towards a new target. ^11^^,^^13^ Utilizing computational modelling platforms to visualize and identify ligand-target interactions is a powerful and efficient approach towards successful drug development. ^14^

This study aims to apply a comprehensive virtual screening approach using multiple platforms to identify drugs and natural products that may potentially be repurposed to inhibit the binding interaction between the SARS-CoV-2 S-glycoprotein and the host ACE2 receptor. Furthermore, the interaction profile of all hit compounds is verified by molecular dynamic simulation studies to provide a detailed insight towards the stability and nature of the binding interaction at the viral-host interface.

## Methods

### Materials and Software

The molecular modelling software Maestro by Schrödinger ^15^ and AutoDock Vina, ^16^ were both used for virtual screening and molecular dynamics simulation studies. The desktop workstation was equipped with Intel® Core™ i7-10700F Processor, Linux Ubuntu 22.10 operating system and a RTX 5000 graphics card.

### Crystal structures

The protein crystal structures were retrieved from the Research Collaboratory for Structural Bioinformatics Protein Data Bank (RCSB PDB) (RRID:SCR_012820). For virtual screening and molecular dynamic simulations, the structure of the SARS-CoV-2 S-glycoprotein receptor binding domain (S-RBD) bound to the ACE2 receptor interface was used (PDB code: 6M0J).

### Virtual databases

A ligand library was compiled comprised of Food and Drug Administration (FDA) approved and worldwide approved drugs as well as approved nutraceuticals and natural compounds with verified *in vivo* efficacy. A total of 1100 FDA approved drugs were retrieved in SDF format from DrugBank database (RRID:SCR_002700), ^17^ along with a library of 3440 worldwide approved medications and 74 approved nutraceuticals. Furthermore, 1537 natural compounds with verified physiological effects in vivo, were retrieved from Zinc database (RRID:SCR_006082). ^18^ Table 1 summarizes the ligand categories of the compiled virtual library.

**Table 1.** Virtual screening library of compounds derived from Drug bank and Zinc online databases.

Library	Number of Compounds	Database	Description
Natural products with experimentally verified in vivo activity	1,537	Zinc	SDF files of approved natural products with in vivo efficacy
FDA approved medications	1,100	DrugBank	SDF files of FDA approved drugs
FDA approved medications	1,614	Zinc	SDF files of FDA approved drugs
Worldwide (not FDA) approved medications	3,440	DrugBank	SDF files of worldwide approved drugs non-FDA
Worldwide (not FDA) approved medications	4,288	Zinc	SDF files of FDA approved drugs
Naturaceuticals	74	DrugBank	SDF files of approved natraceutics
**Final Comprehensive library compiled composed of all compounds listed above, redundant or repeated structures were excluded**	**7,476**	**DrugBank and Zinc**	**SDF files of all FDA and worldwide approved medications and natural products in addition to natural products with verified physiological activity**

### Maestro

*Protein preparation*

All crystal structures were prepared using the Schrödinger Maestro’s (RRID:SCR_016748) protein preparation wizard tool. Structure preparation and minimization was done at a pH 7.4 with corrected ionization states, polar hydrogens were added, and non-essential water molecules were removed. The entire structure was minimized and optimized with the OPLS3 force field and the default value for the RMSD of 0.30 Å was used for non-hydrogen atoms.

*Ligand library preparation*

Downloaded SDF structures were prepared for docking studies using the maestro Ligprep tool (RRID:SCR_016748). Structures were converted into 3D maestro format, and ionization states and chirality were optimized at physiological pH (7.4) using OPLS3 force field. The final 3D conformations were utilized for virtual screening.

*Binding pocket determination and docking studies*

In Schrödinger’s Maestro (RRID:SCR_016748) the binding pocket was identified using Schrödinger’s Sitemap, a single binding pocket between the interface of the RBD and ACE2 binding region (PBD 6M0J) was used. The selected binding pockets were verified by ensuring the presence of essential binding residues identified in previous studies. ^6^ The site score value was within the range of 1-1.1 for each of the binding pockets indicating high accessibility and druggability of the selected binding pocket. The selected binding pockets were used to generate a docking grid using Maestro’s Glide module for docking studies. The receptor grids were generated using the prepared proteins, with the docking grids centered on the identified receptor binding pocket for each protein. A receptor grid was generated using a 1.00 van der Waals (vdw) radius scaling factor and 0.25 partial charge cut-off. The binding sites were enclosed in a grid box of 20 Å ^3^ without constraints and using default parameters. Docking was repeated and verified using three screening settings. All compounds were screened under a high-throughput docking setting and the top 200 compounds with the highest binding scores where then selected for standard precision docking, of these verified hits the top 80 compounds where further verified using extra precision (XP) docking settings. The ligands were docked using the extra precision mode (XP) without using any constraints and a 0.80 van der Waals (vdw) radius scaling factor and 0.15 partial charge cut-off. Induced fit docking was carried out with flexibility of the residues of the pocket near to the ligand.

GlideScore implemented in Glide (RRID:SCR_016748), was used to estimate binding affinity and rank ligands. The XP Pose Rank was used to select the best-docked pose for each ligand. The final list of thrice verified compounds was then analyzed in detail based on binding scores and a detailed study of all binding interactions.

*Molecular dynamics simulation studies*

Molecular dynamic (MD) simulation studies were carried out using the Desmond Module on Schrödinger’s Maestro platform (RRID:SCR_016748). The protein preparation wizard was used to minimize the hit protein-ligand complex and the simulation environment was built using the system builder application of Desmond. A water based solvent system: TIP3P was employed to generate the simulation environment contained within an orthorhombic simulation box with 10 Å buffer parameter from the protein surface. The system was neutralized and isotonic conditions attained via the addition of counter ions and 0.15 M NaCl. All MD simulations were conducted at a temperature of 300 K and a pressure of 1.013 bar. A simulation period of 100 nanoseconds was run for each of the hit ligand-protein complexes. Analysis calculations were subsequently run and results presented using the simulation interaction diagram tool of Desmond.

### Autodock vina

Docking calculations were carried out using the AutoDock vina software version 1.1.2 (RRID:SCR_011958). ^16^ All hydrogens were added to the ligand PDB file and Gasteiger charges were computed and all the torsion angles of the ligand were defined using the autodock-tools program. A grid box generated with the following dimensions: 36×24×-4 Å, with a grid spacing of 1 Å was used. The Lamarckian genetic algorithm was used as a search method with a total of 30 runs (maximum of 20 000 000 energy evaluations; 27 000 generations; initial populations of 150 conformers). The binding affinity calculation in AutoDock vina together with analysis of binding interactions were used to select hits for molecular dynamic simulation studies.

## Results

### Docking studies

Preliminary docking studies to identify ligands that interact favorably with the receptor binding domain of the spike protein, involved screening a total of over 7000 approved medications and natural compounds (see Table 1). Initial docking studies were carried out on the S-RBD and ACE2 interface using Schrödinger Glide and Autodock Vina to identify hits that would prevent/disrupt the crucial protein-protein interaction. The results from both programs were analyzed and compared. The compounds that showed the most favorable binding profile and the best binding scores were shortlisted in Table 1. ^29^^,^^30^

**Figure 1.**
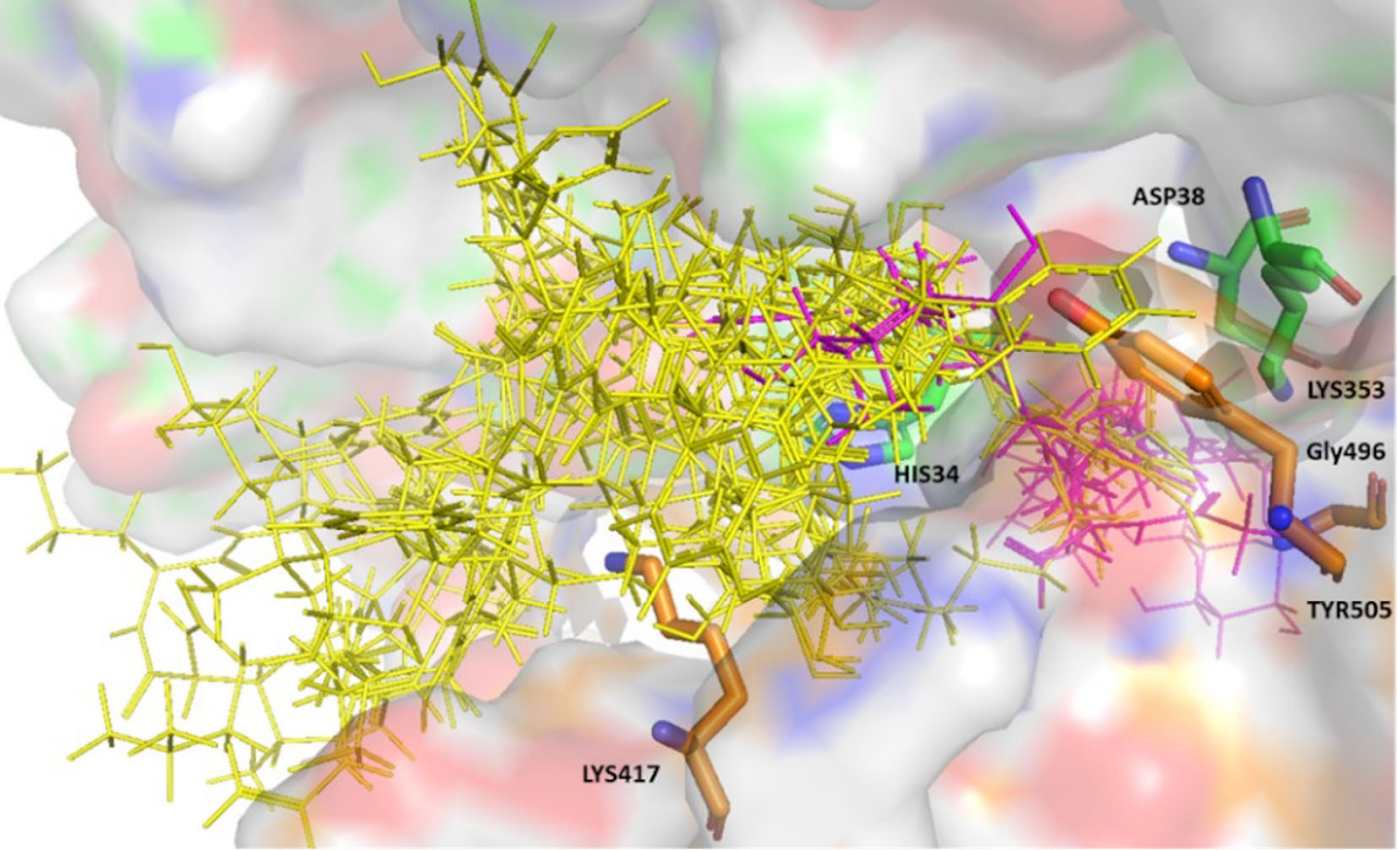
Surface representation of the 13 hits bound to the ACE-2 S-glycoprotein interface. ACE-2 residues with carbon in green and S-glycoprotein residues with carbon in brown. The hit compounds are represented in yellow with hexoprenaline, n-acetyglucosamine, and tricocin highlighted in magenta.

From the initial hits 13 were identified that showed consistent good binding scores and binding poses. The compounds shortlisted bound to the protein-protein interface, and established interactions with key residues namely, His34, Asp38, and Lys353 from the ACE-2 binding region and Lys417, Gly496 and Tyr505 from the S-RBD. Hexoprenaline and tricocin, being highly flexible molecules and n-acetylglucosamine being a relatively small molecule were able to burrow deeply within the protein interface, while maintaining interactions with the above-mentioned key residues ( Figure 1). The hit compounds all expressed high binding affinity scores using both platforms; ranging from -7.5 — -12.4 (Glide scoring), and -4.8 — -8.9 (Autodock vina scoring).

**Figure 2.**
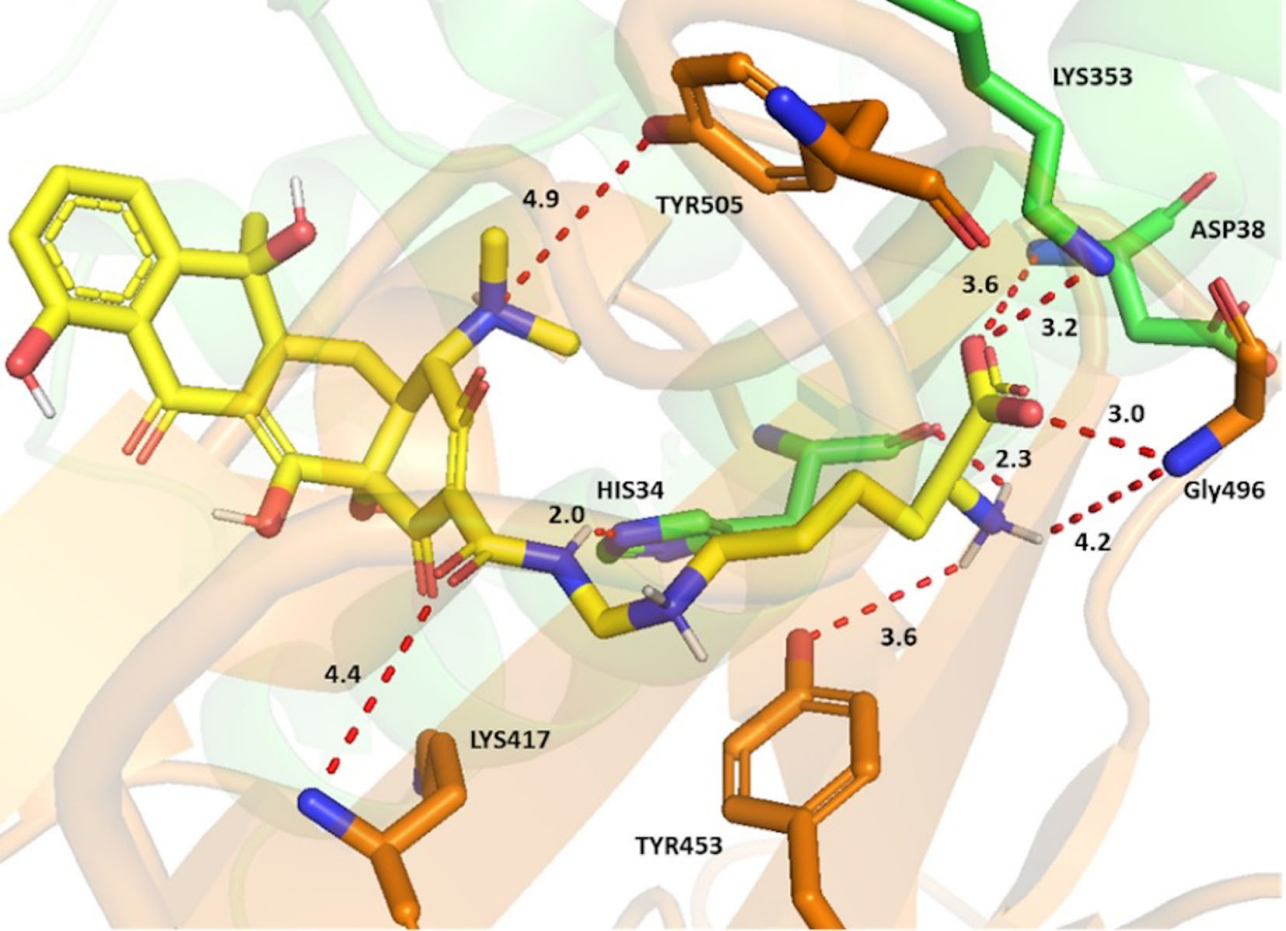
Binding interactions of lymecycline with the ACE-2 S-glycoprotein interface. ACE-2 residues with carbon in green and S-glycoprotein residues with carbon in brown. Potential electrostatic interactions are represented as red dotted lines and distances are in Angstrom.

Lymecycline, as an example, established key interactions *via* its side chain groups ( Figure 2). The carboxylate formed a crossbridge at the interface; with strong charge assisted H bond between the Asp38 and Lys353 sidechains from ACE-2 and the Gly496 sidechain from the spike protein. The interaction was further strengthened by the ammonium ion of the terminal amino acid forming a charge assisted H bond with both the His34 backbone (ACE2) and Gly496 sidechain (S-RBD). A water mediated H bond for this ammonium ion with the Tyr453 sidechain of the S-RBD was also observed. Potential H bonds were also noted between the sidechain amide oxygen and Lys417, as well as the ring’s tertiary amine with Tyr505 from the spike protein. An increase of selectivity for lymecycline is expected owing to the presence of two opposite electrostatic interactions with both chains. The first a H bond between the sidechain amide and His34 sidechain (ACE-2) and the second is a H-π interaction between the central aliphatic ammonium and Tyr453 aromatic ring (S-RBD).

### MD simulation results

**Figure 3.**
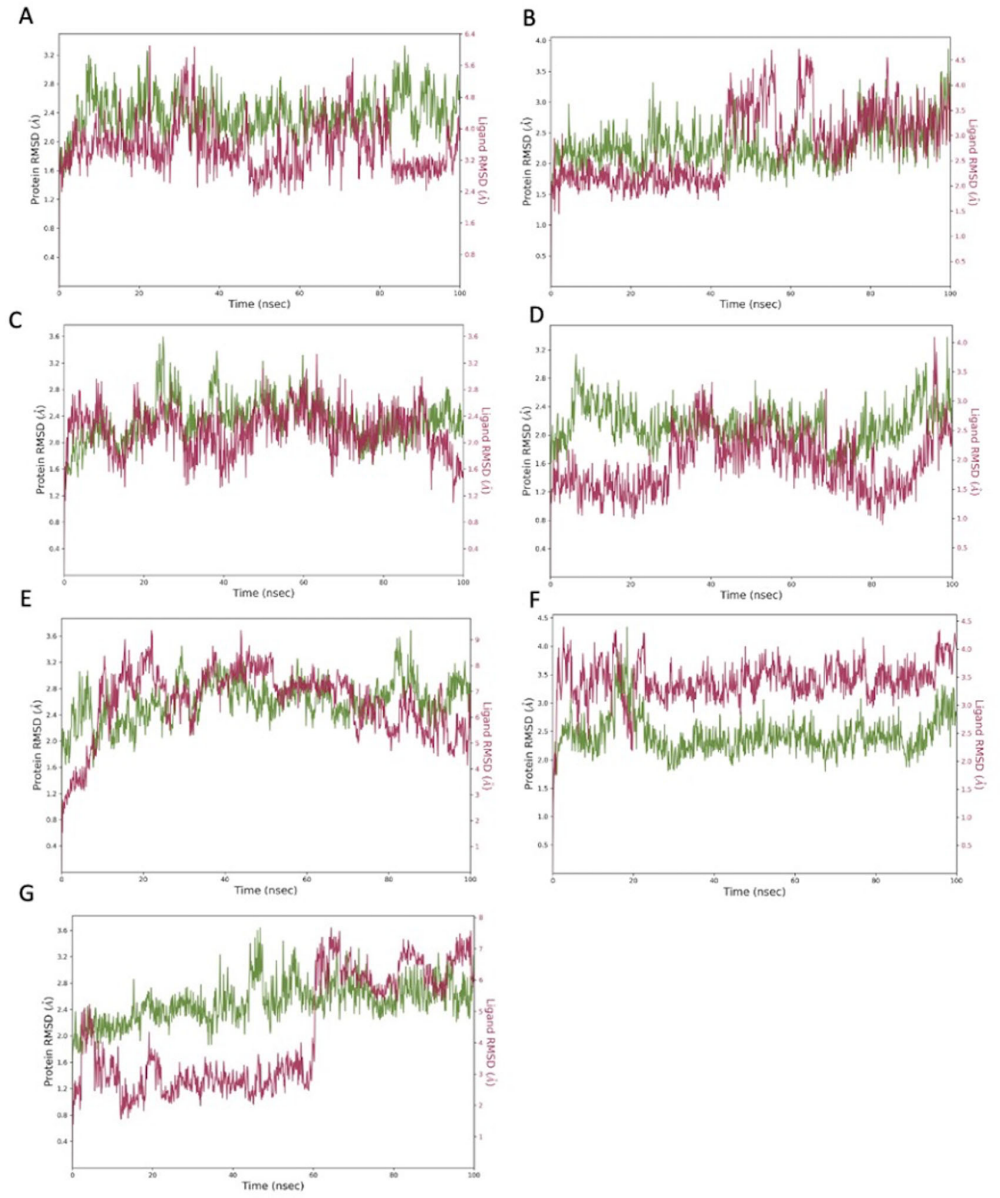
Root mean square de *via*tion (RMSD) graphs for the top hit compounds A: Lymecycline, B: Hexoprenaline, C: Pentagalloylglucose, D: Polydatin, E: Tricrocin, F: Setmelanotide, G: Forsythiaside. The green graph shows fluctuations in the protein backbone from the initial reference point while the red shows the ligand fluctuations. The RMSD profile of the ligand is with respect to its initial fit to the protein binding pocket indicates that all ligands did not fluctuate beyond a 2-4 Å range.

**Figure 4.**
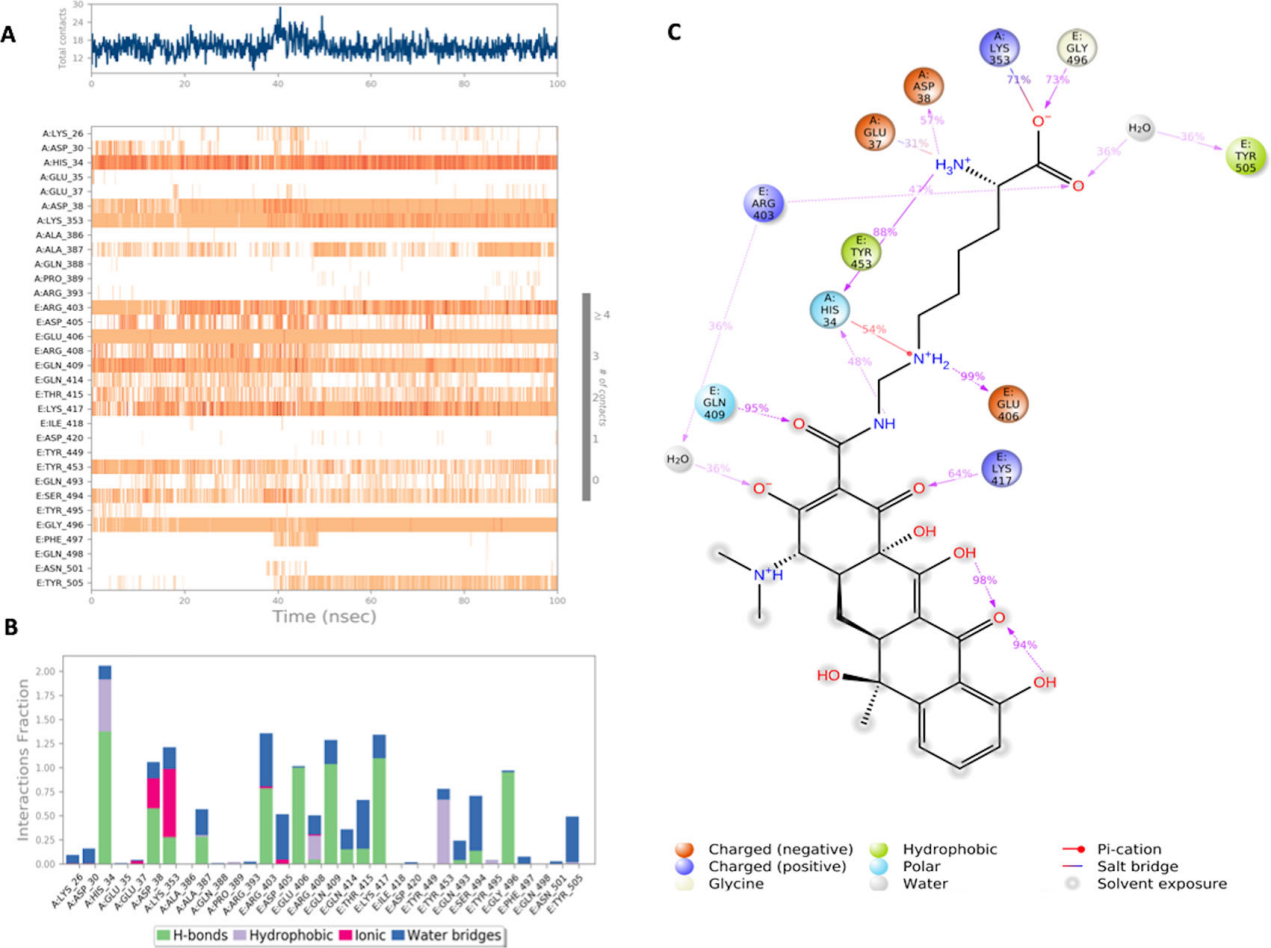
Interaction diagram of Lymecycline with S-RBD-ACE2 interface binding pocket. (A) Interaction of lymecycline with residues in each trajectory frame. The depth of color indicating the higher the interaction with contact residues; (B) The protein-ligand contacts showing the binding interactions fraction; (C) Lymecycline interactions with the protein residues during MD simulation. Interactions shown are occurring more than 30% during the simulation time. A: chain A of the ACE2 binding domain, E: chain E of the S-glycoprotein receptor binding domain.

**Figure 5.**
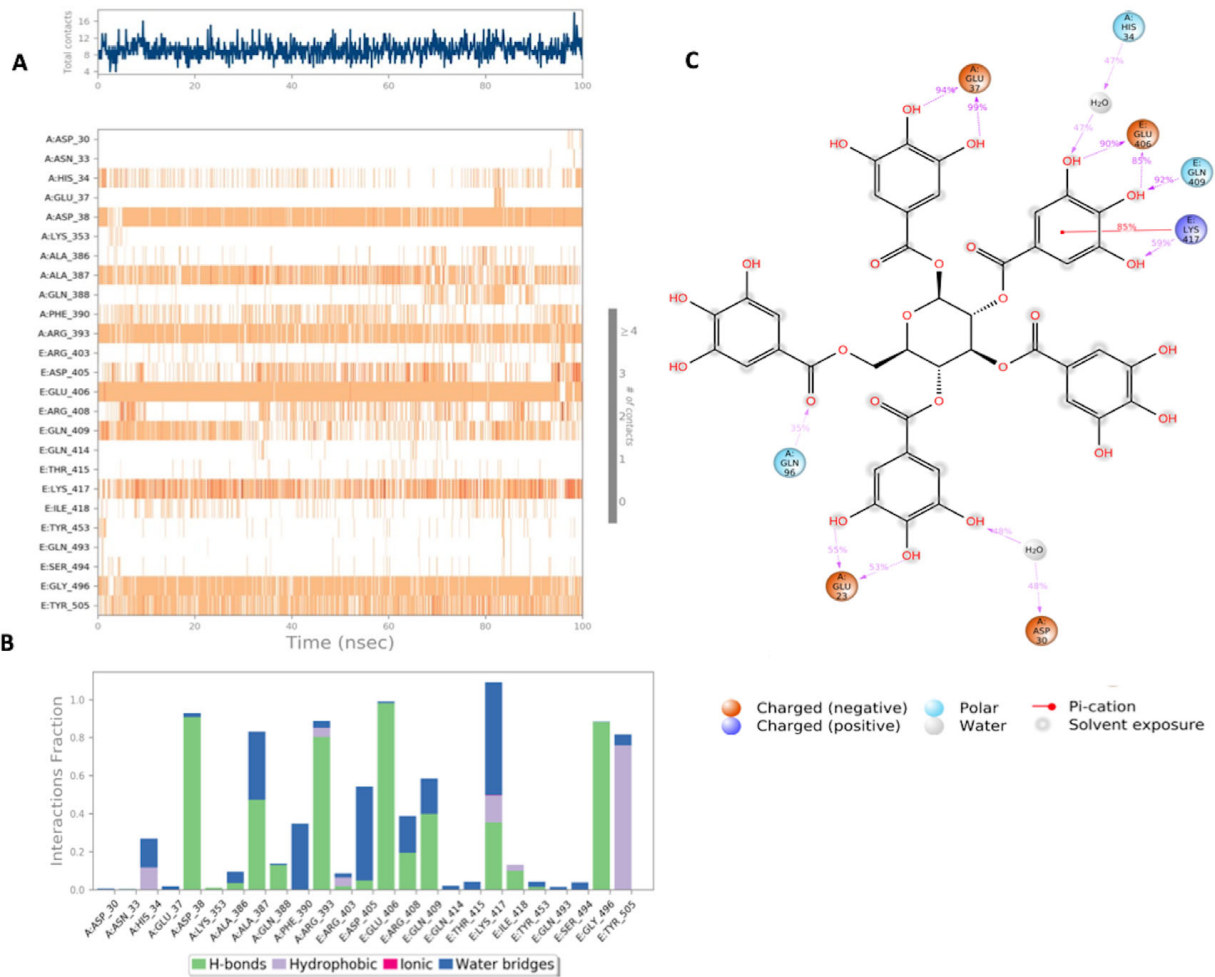
Interaction diagram of Pentagalloylglucose with S-RBD-ACE2 interface binding pocket. (A) Interaction of Pentagalloylglucose with residues in each trajectory frame. The depth of color indicating the higher the interaction with contact residues; (B) The protein-ligand contacts showing the bonding interactions fraction; (C) Pentagalloylglucose interaction with the protein residues during MD simulation. Interactions shown are occurring more than 30% during the simulation time.

**Figure 6.**
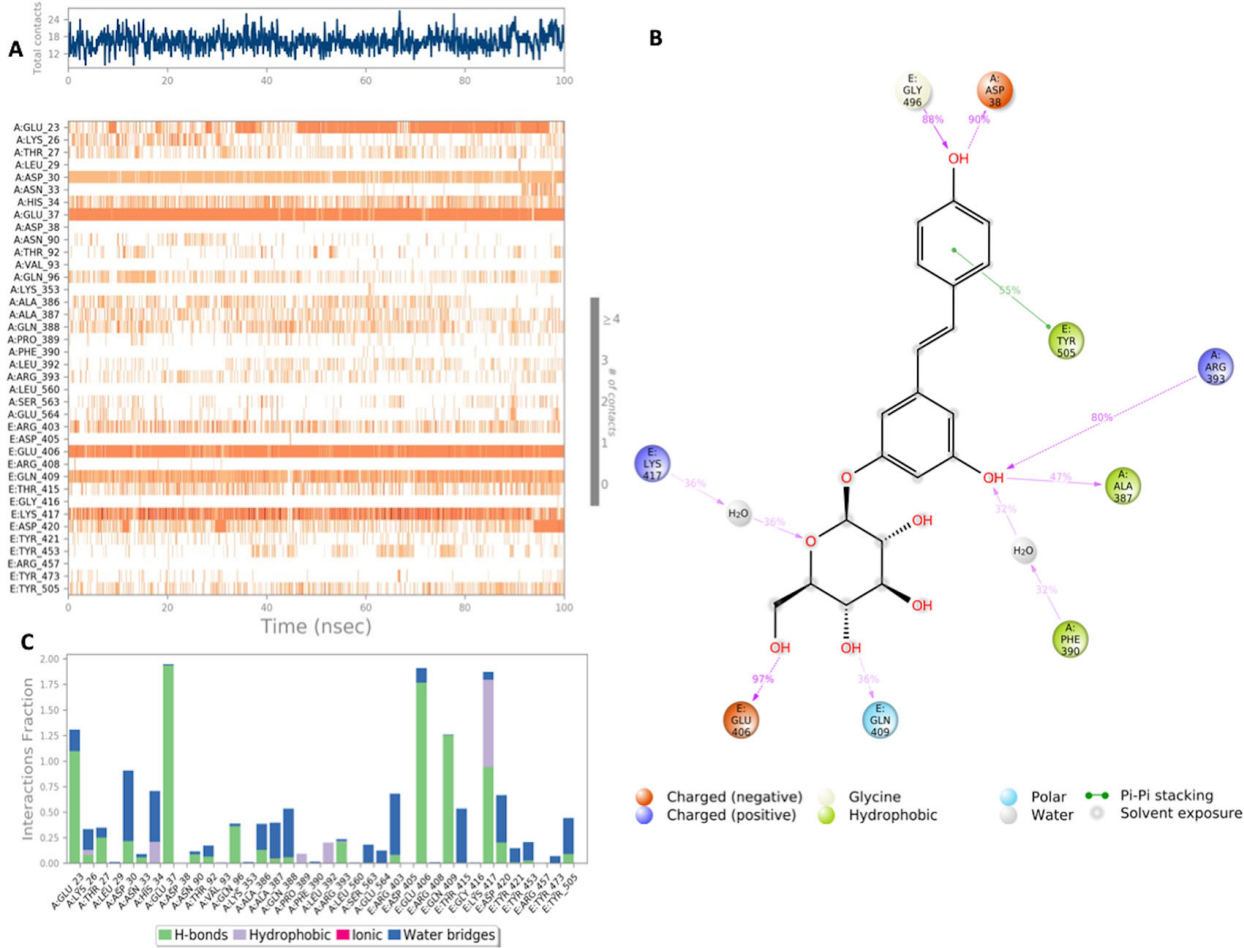
Interaction diagram of polydatin with S-RBD-ACE2 interface binding pocket. (A) Interaction of polydatin with residues in each trajectory frame. The depth of color indicating the higher the interaction with contact residues; (B) The protein-ligand contacts showing the bonding interactions fraction; (C) Polydatin interaction with the protein residues during MD simulation. Interactions shown are occurring more than 30% during the simulation time.

The top hit molecules were selected based on a detailed visual analysis of the interaction profile of each compound with the target proteins. A hit was identified as any compound which had a favorable binding score and profile. Compounds were shortlisted based on reproducibility of the results using two different docking platforms (Glide and AutoDock Vina). 13 hit ligands were selected for molecular dynamic simulation studies ( Table 2). Investigation of the optimal 2D and 3D docked positions reveled that each of the hit ligands form interactions with key residues of the binding pocket. Root mean square deviation (RMSD) plot analysis was used to measure the average displacement of atoms with respect to a reference frame. RMDS analysis revealed a stable binding profile for the top 7 ligands as shown in Figure 3 (RMSD fluctuation range of 2-4 Å). Of the 7 hit ligands, 3 exhibited a highly stable and robust binding profile within the interface binding pocket, these include: lymecycline, pentagalloylglucose and polydatin. The interaction profile for each individual ligand is depicted in Figures 4- 6 (for MD results for the remaining 4 ligands see extended data). As the S-RBD-ACE2 interface structure was used for simulations, all interactions of the ligand with key binding residues of both chain A of the ACE2 binding domain and chain E of the S-RBD were considered as significant.

**Table 2.** The individual docking scores for the hit molecules using both AutoDock Vina and Schrödinger’s Glide at the interface of the S-glycoprotein and ACE2 receptor PDB 6M0J.

Ligand	Binding affinity (kcal/mol) Software: Schrödinger Glide	Binding affinity (kcal/mol) Software: AutoDock Vina
Colistin	-12.4	-4.8
Argipressin (vasopressin)	-9.7	-6.8
Lymecycline	-9.1	-8.3
Setmelanotide	-9.0	-6.1
Polydatin	-7.6	-8.5
Plazomicin	-10.6	-7.3
Hexoprenaline	-10.1	-6.5
Tricrocin	-9.1	-8.4
Ginsenosides	-11.3	-8.0
Ademetionine	-7.6	-7.4
N-Acetyl-D-Glucosamine	-7.5	-6.0
Pentagalloylglucose	-12.7	-8.9
Forsythiaside	-11.7	-8.7

Lymecycline shows an exceptional binding profile with key binding residues of the binding pocket as shown in Figure 4. Key binding residues of the S-RBD Lys417 and Gly496 form a H bonding interaction with the lymecycline, furthermore it exhibited water bridge interactions with Tyr 505 and Ser494. While at the interface lymecycline was also shown to form interactions with key binding residues of the ACE2 binding domain including a strong H bond with His34 and mixed interactions (ionic and hydrogen bonds) with residues Asp38 and Lys353. All significant interactions are represented in Figures 4 A and B, which highlight residues with the strongest ligand interactions that are stable over the entire simulation period. Figure 4 C depicts all significant interactions displayed by the ligand and interacting residues occurring for over 30% of the simulation period. Of significance Lys417 and Gly496 are bound to lymecycline over 60% of the simulation period.

MD simulation results for pentagalloylglucose in Figure 5 show significant interactions with key residues of the binding pocket. Key binding residues of the S-RBD: Lys417 and Gly496, form H bonds, and Lys417 was also shown to form a water bridge interaction with the ligand. Additionally, a stable hydrophobic interaction with residue Tyr505 was observed. Strong interactions with key residues of the ACE2 binding domain were also observed in the simulation period, notably H bonds and water bridges with residues Asp38 and Arg393. All significant interactions are represented in Figures 5A‐C. Of significance Lys417 interacts with pentagalloylglucose approximately 85% of the simulation period.

Polydatin exhibits several stable interactions throughout the 100 nanosecond simulation period as depicted in Figure 6. Polydatin binds with key residues of the S-RBD Lys417 and Tyr505. A mixed binding profile is observed including H bond interactions, water bridges and for Lys417 hydrophobic interactions. Polydatin was also shown to form interactions with key residues of the ACE2 binding domain including a strong hydrogen bond with His37 and mixed H bond and water bridge interaction with residue Asp30. All significant interactions are represented in Figure 6.

## Discussion

Over the last two decades, different docking tools and programs have been developed that use different algorithms in which the conformation of the ligand is extensively evaluated in a binding pocket until an energy minimum is reached. Most programs treat the ligand as a flexible component and the receptor as rigid while others treat both interacting components (ligand and receptor) as flexible. ^16^ Such programs not only differ in the type of docking, but also in their ligand placement strategies. ^19^ In this study, we selected Schrödinger’s Maestro and Autodock vina in order to assess the docking accuracy and mode of binding. Autodock vina was used to perform rigid docking while maestro was used for induced fit (flexible) docking. Autodock uses the genetic algorithm while maestro uses systematic search techniques for ligand placement.

This study was a comprehensive *in silico* investigation of a key target of the SARS-CoV-2 virus; the surface S-glycoprotein or spike protein. The interaction between the S-glycoprotein and target ACE2 host receptor is essential to the virulent nature of the virus. Agents that can disrupt this binding interaction may potentially inhibit viral entry into the host cell and infection. Structures selected for this study were all solved by x-ray crystallography or EM with a minimum accepted resolution of 2.45 Angstrom. For each of the target structures, the selected binding pockets from Schrödinger’s Sitemap were verified by ensuring the presence of essential binding residues identified in previous studies ( Table 2). A detailed study of the viral host interface depicted in the 6M0J crystal structure revealed the key residues involved in strong charge assisted H bonds, ionic bonds, and strong H bonds, judged by the bond length, charge, and orientation ( Table 3). Binding to these residues is necessary to disrupt the natural interaction between the two proteins in favor of the hit ligand.

**Table 3.** Key and allosteric binding residues of the binding pocket.

Protein name	Key binding residues	Allosteric binding residues	Reference
SARS-CoV-2 S glycoprotein (spike protein)	Lys417, Gly446, Leu455, Tyr449, Tyr453, Phe456, Phe486, Asn487, Tyr489, Gln493, Gly496, Gln498, Thr500, Asn501, Gly502, Tyr505	Gly488 Gly502 Asp427 Asp428 Lys986 Lys386 Leu387 Asp614	^6^^,^^27^^,^^28^
Angiotensin converting enzyme-2 (binding interface)	Gln24, Thr27, Phe28, Asp30, Lys31, His34, Glu35, Glu37, Asp38, Tyr41, Gln42, Leu79, Met82, Tyr83, Asn330, Lys353, Gly354, Asp355, Arg357, Arg393		^6^^,^^27^

In our study, a comprehensive screening library of over 7000 compounds was compiled, comprised of both FDA and worldwide approved drugs and nutraceuticals in addition to all natural products with established *in vivo* activity (Zinc) ^18^ (Drugbank). ^17^ In order to ensure robust and reproducible results virtual screening was preformed using two different platforms: Glide ^15^ and Autodock Vina. ^16^ The thorough protocol employed within this study ensures that the hit ligands identified had verified binding profiles. Further molecular dynamic simulation studies were vital towards verifying and visualizing the nature of the binding interaction of the hit ligands within the binding interface. Hits, that were selected for MD simulation studies, were determined to be the most likely to disrupt the strongest interactions between the viral and host proteins ( Table 2).

MD simulation studies identified the following hit ligands: lymecycline, hexoprenaline, pentagalloylglucose, polydatin, tricrocin, setmelanotide and forsythiaside. All hits expressed a stable interaction profile as indicated by RMSD below 4 Å for both proteins and ligand position. It is important to highlight that all compounds were shortlisted from initial screening results based not only on their binding profile but also their suitability to be translated towards a clinical setting. Several drugs used in the management of COVID-19 have had detrimental effects owing to the adverse drug reaction profile of the employed therapeutic. The pathogenesis of COVID-19 culminates in several immune and cardiovascular manifestations ranging from hypercoagulability to kidney failure, as of such agents that are not appropriately selected may cause more harm than benefit within the overall scope of disease management. ^20^ Four hits were selected including lymecycline, pentagalloylglucose, polydatin, and hexoprenaline, which expressed very good RMSD profiles, indicating that the ligand remained bound in a stable manner within the binding pocket throughout the entirety of the simulation period. Other shortlisted ligands such as setmelanotide, and forsythiaside appear to have fluctuated with respect to their initial position relative to the protein backbone at some point within the simulation period although, the range of the fluctuation did not exceed beyond the range of 2-4 Å. The three final hits; lymecycline, pentagalloylglocose and polydatin, maintain strong bonds with residues within the binding pocket and in some cases vital binding residues throughout the entire simulation period.

Lymecycline, a broad-spectrum second-generation tetracycline antibiotic commonly used in the management of acne, gynecological and respiratory tract infections, was shown to exhibit stable binding with key binding residues of both the spike and ACE2 RBD. Lymecycline maintained its interaction with its side chain throughout the simulation period. The stable charge assisted H bond with Lys 353 from ACE-2 and Gly 496 from spike was conserved during the dynamic simulation which suggests that these interactions are more energetically favored over the initial H bond between the two mentioned residues. Other crucial interactions are shown with residues; Glu 37 and Asp 38 from ACE-2 and Tyr 505 from the S-RBD. These residues were bound *via* a H bond in the original protein-protein interaction. Lymecycline has been reported in previous studies to bind to additional viral targets in the SARS-CoV-2 virus, namely the main protease (M ^pro^). ^21^ Like many members of its class it expresses anti-inflammatory properties, ^22^ this makes it a particularly attractive subject that can be further investigated for repurposing studies in the treatment of SARS-CoV-2.

Pentagalloylglucose is a polyphenolic compound that has been shown in *in vitro* and *in vivo* studies to have anti-viral effects against the hepatitis C virus. ^23^ A number of recent *in vitro* studies have shown a dose dependent effect in inhibiting viral spike association with the host ACE2 receptor. ^24^ Our study reveals the detailed nature of the binding interaction of pentagalloylglucose at the S-RBD-ACE2 binding interface. Key residues Lys417, Gly496 and Tyr505 of the viral spike protein and residues Asp38 and Arg393 of the ACE2 binding domain, form a stable binding interaction with pentagalloylglucose. Interference with viral-host binding at these key residues is very likely to be vital for the inhibition of viral engagement with the host ACE2 receptor and subsequent cell entry.

Polydatin, another polyphenolic compound, is a glycoside precursor of resveratrol. *In vitro* studies have shown its potential to inhibit viral spike protein binding with the ACE2 receptor. ^25^ The findings of this study corroborate our own where similar binding scores were reported. Furthermore, our studies identify strong interactions of the key binding residues Lys417 of the S-RBD and Asp30 and Glu37 of the ACE2 binding region with polydatin. It appears that interfering with the binding of the viral Lys417 residue is a key inhibitory pattern detected in a number of docking profiles supported by *in vitro* findings. ^24^^,^^25^

Hexoprenaline, a β2 adrenoceptor agonist, is mentioned as a drug of interest although it was not identified from the top three- hits that showed the most stable interactions during MD simulations. It is for the first time reported to exhibit a favorable binding profile with a number of key binding residues of the S-glycoprotein RBD as well as the ACE2 binding domain. Hexoprenaline was shown to bind key residues and to interrupt key interactions. One of its side-chain nitrogen atoms formed two charge assisted H bonds with the two initially bound residues Glu35 from ACE-2 and Gln493 from S-RBD. The other chain nitrogen formed a charge assisted H bond with Glu37 (see extended data for MD results of remaining shortlisted compounds). The stability of these critical bonds during dynamics was slightly lower than those noted for lymecycline during the simulation period. Yet, considering its role as a bronchodilator and its pharmacological profile it may be considered a good candidate for drug repurposing in the management of COVID-19 infection where there is a high incidence of respiratory distress. However, caution must be taken, considering its potential nonselective activity on β1 receptors which may result in unwanted cardiovascular effects. ^26^

In this study lymecycline, pentagalloylglucose, and polydantin were identified as potential inhibitors of the S-RBD-ACE2 binding interface. Hexoprenaline could be also considered as a promising hit, due to its favorable docking and dynamic profile and taking into account its relevance and suitability for clinical testing. Of the nutraceuticals, forsythiaside also appears promising in its ability to potentially disrupt key binding interactions at the viral-host interface and is a prime candidate for further *in vitro* and *in vivo* studies. From a clinical perspective, both lymecycline and hexoprenaline may be considered as possible candidates for preliminarily clinical studies to assess their therapeutic potential in the management of COVID-19 infections.

## Conclusions

Virtual screening is a highly attractive approach to identify potential compounds with therapeutic efficacy against target proteins, while efficient, it is not without its limitations. There have been a large number of docking studies published in the literature that have identified agents that may potentially be repurposed to inhibit SARS-CoV-2 targets. Our study is unique in its comprehensive approach to identify agents that can bind to the viral S-glycoprotein-ACE2 binding interface using multiple platforms. Molecular dynamic simulation studies were essential to identify a consistent binding pattern that appears to be common in the most effective agents that have the potential to inhibit the S-glycoprotein-ACE2 interaction. The agents identified in this study were additionally shortlisted for their suitability to be translated to a clinical COVID-19 setting by understanding their toxicity profile and identifying agents with verified anti-inflammatory and anti-viral capacity. The hit compounds identified are prime agents for further *in vitro* and clinical investigations to verify their efficacy in the potential treatment of COVID-19.

## Author contributions

D.H. designed the research project. D.H., M.G., A.A., and A.H. conducted the computational studies. D.H. and M.G. analyzed the results. S.M., H.A., M.A., helped draft the manuscript. All authors were involved in writing, editing and revision of the manuscript.

## Data Availability

Source data include the protein crystal structures which were retrieved from the Research Collaboratory for Structural Bioinformatics Protein Data Bank (RCSB PDB) (RRID:SCR_012820). http://www.rcsb.org/#Category-welcome . The PDB accession code for the protein crystal structure of the S-glycoprotein bound to the ACE-2 receptor is 6M0J, and the pdb sturcture file can be accessed here: https://www.rcsb.org/structure/6m0j. Additional source data include the ligand libraries which were retrieved from online databases: DrugBank database (RRID:SCR_002700) found here: http://www.drugbank.ca/. Annual account subscription and access to ligand libraries was kindly provided for the purpose of academic research. Freely available ligand libraries were downloaded from the open access Zinc database (RRID:SCR_006082) found here http://blaster.docking.org/zinc/. See Table sd1 below and/or in Table 1 in the main text for complete details. Figshare: RAW DATA FILE: In silico drug repurposing using molecular docking and dynamics to target the protein interaction between the SARS-CoV -2 S-glycoprotein and the ACE2 receptor. https://doi.org/10.6084/m9.figshare.22257352.v5. ^30^ This project contains the following underlying data: Simulation results: final trajectory frames of the hit compounds bound to the protein interface:
-
ligprep_DrugBannkApprovedNOMETAL28072021_maegz_1967.pdb -
ligprep_DrugBannkApprovedNOMETAL28072021_maegz_2048.pdb -
ligprep_DrugBannkApprovedNOMETAL28072021_maegz_135.pdb -
ligprep_DrugBannkApprovedNOMETAL28072021_maegz_7.pdb -
ligprep_DrugBannkApprovedNOMETAL28072021_maegz_654.pdb -
ligprep_WorldappNOTFDAZinc_maegz_3777.pdb -
ligprep_DrugBannkNaturaceuticalNOMETAL28072021_2_maegz_83.pdb -
ligprep_DrugBannkNaturaceuticalNOMETAL28072021_2_maegz_5.pdb -
ligprep_DrugBannkNaturaceuticalNOMETAL28072021_2_maegz_27.pdb -
ligprep_NaturalINvivoZinc_NOMETAL_maegz_1403.pdb -
ligprep_NaturalINvivoZinc_NOMETAL_maegz_1472.pdb -
ligprep_WorldappNOTFDAZinc_maegz_3450.pdb -
ligprep_WorldappNOTFDAZinc_maegz_40.pdb Protein structure:
- spikeACE2RBDinterface_protein.pdb Ligand libraries derived from open-source databases:
- FDAapproved medications.sdf - world-not-fdaApproved medication.sdf - NaturalcompoundsLibrary.sdf Extended Data: In silico drug repurposing using molecular docking and dynamics to target the protein interaction between the SARS-CoV -2 S-glycoprotein and the ACE2 receptor. https://doi.org/10.6084/m9.figshare.22414234.v1. ^30^ This project contains the following extended data:
- Extended Data Insilico Drug repurposing SglycoproteinACE2.docx Data are available under the terms of the Creative Commons Attribution 4.0 International license (CC-BY 4.0).
