# Supplementary material for: In silico drug repurposing using molecular docking and dynamics to target the protein interaction between the SARS-CoV-2 S-glycoprotein and the ACE2 receptor
Source: F1000Res. 2024 Jul 26;12:1452. Originally published 2023 Nov 9. [Version 2] doi: 10.12688/f1000research.131508.2 (PMC11519612; doi:10.12688/f1000research.131508.2)
Supplement: Peer review history

# Reviewer report: Reviewer response for version 2

**DOI:** 10.5256/f1000research.169234.r307418

**Published:** 2024-10-28

**Authors:** Dr Manne Munikumar

**Affiliations:** 1 ICMR-National Institute of Nutrition, Hyderabad, Telangana,, India

Dear Author,

 Your revised manuscript, entitled "In Silico Drug Repurposing Using Molecular Docking and Dynamics to Target the Protein Interaction Between the SARS-CoV-2 S-Glycoprotein and the ACE2 Receptor," may be considered for indexing in the esteemed journal.

Is the work clearly and accurately presented and does it cite the current literature?

Yes

If applicable, is the statistical analysis and its interpretation appropriate?

Yes

Are all the source data underlying the results available to ensure full reproducibility?

Yes

Is the study design appropriate and is the work technically sound?

Yes

Are the conclusions drawn adequately supported by the results?

Yes

Are sufficient details of methods and analysis provided to allow replication by others?

Yes

Reviewer Expertise:

Molecular Docking, Bioinformatics

I confirm that I have read this submission and believe that I have an appropriate level of expertise to confirm that it is of an acceptable scientific standard.

# Reviewer report: Reviewer response for version 2

**DOI:** 10.5256/f1000research.169234.r317619

**Published:** 2024-09-10

**Authors:** Vipul Kumar, Vikas Tiwari

**Affiliations:** 1 Bioengineering and Therapeutic Sciences, University of California San Francisco, San Francisco, California, USA

Authors have conducted an extensive screening and modeling study. However, I suggest addressing the points below to enhance the paper:

 1. The binding sites were enclosed in a grid box of 20 Å³ without constraints using default parameters." (The grid box size of 20 Å³ seems very small and needs verification.)

 2. The final complex configuration from compiled trajectories of a 100 ns MD simulation was used to calculate the MMGBSA free energy. (Consider calculating across the trajectory or using cluster representatives.)

 3. Upon designing the study and to this date there are no known solved crystal structures for the SARS-Cov-2 RBD and ACE2 complex bound to a ligand within the interacting binding pocket, as of such for the purpose of validation two different docking platforms were employed in the initial docking investigations to screen and shortlist suitable candidates targeting our region of interest in the viral-host interface. (Improve the sentence)

 4. The Gly496 from the spike protein" (Glycine does not have a side chain. Refer to it as the Gly496 backbone)

 5. "RMSD analysis revealed a stable binding profile for the top 7 ligands as shown." (RMSD, not RMDS.)

 6. The side effects of the top hits should be discussed.

 7. All hits expressed a stable interaction profile as indicated by RMSD below 4 Å for both proteins and ligand position." (Not correct as per the Figure 3)

 8. Can these compounds bind with a similar score to individual ACE2 or spike proteins alone?

 9. Are there other docking studies targeting ACE2-spike interactions? If so, the top hits from those studies could be used for comparison."

Is the work clearly and accurately presented and does it cite the current literature?

Yes

If applicable, is the statistical analysis and its interpretation appropriate?

Partly

Are all the source data underlying the results available to ensure full reproducibility?

Yes

Is the study design appropriate and is the work technically sound?

Yes

Are the conclusions drawn adequately supported by the results?

Partly

Are sufficient details of methods and analysis provided to allow replication by others?

Partly

Reviewer Expertise:

Molecular Modelling and Computational Drug Discovery.

We confirm that we have read this submission and believe that we have an appropriate level of expertise to confirm that it is of an acceptable scientific standard, however we have significant reservations, as outlined above.

# Reviewer report: Reviewer response for version 1

**DOI:** 10.5256/f1000research.144356.r260767

**Published:** 2024-05-20

**Authors:** Dr Manne Munikumar

**Affiliations:** 1 ICMR-National Institute of Nutrition, Hyderabad, Telangana,, India

**Dear Authors,**

 The manuscript entitled “In silico drug repurposing using molecular docking and dynamics to target the protein interaction between the SARS-CoV-2 S-glycoprotein and the ACE2 receptor.” thoroughly assessed. The manuscript demonstrates a rigorous methodology, employing two different computational platforms for virtual screening, molecular docking, and molecular dynamics simulations. This comprehensive approach enhances the reliability and robustness of the findings. The study addresses an important and timely topic by focusing on the protein interaction between the SARS-CoV-2 S-glycoprotein and the ACE2 receptor, which is crucial for viral entry and infection. The potential for drug repurposing strategies to combat COVID-19 is underscored, highlighting the significance of the research. The manuscript successfully identifies several potential hit compounds with stable interactions at the viral-host interface. The selection of lymecycline, pentagalloylglucose, polydatin, and hexoprenaline for further investigation demonstrates promising results and provides a basis for future experimental studies. The study provides valuable insights into the binding mechanisms of the identified hit compounds, elucidating their interactions with key viral and host residues. This deepens understanding of the molecular basis of SARS-CoV-2 infection and potential therapeutic interventions. The conclusion offers practical implications by suggesting lymecycline and hexoprenaline as candidates for preliminary clinical studies. This highlights the translational potential of the findings and their relevance for the development of COVID-19 therapeutics. Following comments has to be addressed.

- While the computational approach is thorough, experimental validation of the findings is lacking. Incorporating in vitro or in vivo studies to confirm the efficacy of the identified compounds would strengthen the manuscript and provide more robust evidence for their potential as therapeutics.
- The manuscript could benefit from a more comprehensive discussion of the limitations and assumptions of the computational methods employed. Addressing factors such as binding site flexibility, solvent effects, and conformational changes could enhance the interpretation of the results and their relevance to real-world scenarios.
- The study focuses on a specific set of compounds and interactions, which may limit its generalizability to other drug candidates or viral strains. Discussing the broader implications of the findings and potential applicability to related viruses or drug targets would enrich the manuscript.
- It would be beneficial for the authors to provide information on the availability of the datasets, software codes, and molecular structures used in the study. Enhancing data accessibility would promote transparency and reproducibility in computational drug discovery research.
- While the selected compounds show promise in silico, their clinical feasibility and safety profiles need to be thoroughly evaluated. Discussion of potential side effects, pharmacokinetic properties, and drug interactions would provide a more comprehensive assessment of their suitability for clinical trials.

Is the work clearly and accurately presented and does it cite the current literature?

Yes

If applicable, is the statistical analysis and its interpretation appropriate?

Yes

Are all the source data underlying the results available to ensure full reproducibility?

Yes

Is the study design appropriate and is the work technically sound?

Yes

Are the conclusions drawn adequately supported by the results?

Yes

Are sufficient details of methods and analysis provided to allow replication by others?

Yes

Reviewer Expertise:

Molecular Docking, Bioinformatics

I confirm that I have read this submission and believe that I have an appropriate level of expertise to confirm that it is of an acceptable scientific standard, however I have significant reservations, as outlined above.

# Response

**Published:** 2024-07-15

**Authors:** dania hussein

**Affiliations:** Imam Abdulrahman bin Faisal University, Saudi Arabia

*We thank the esteemed reviewer for his valued input and comments. We have addressed and responded to each comment accordingly:*

  

- While the computational approach is thorough, experimental validation of the findings is lacking. Incorporating in vitro or in vivo studies to confirm the efficacy of the identified compounds would strengthen the manuscript and provide more robust evidence for their potential as therapeutics.

*We thank the reviewer for this valuable comment. The current research involves in silico drug repurposing for COVID-19 through identifying potential ligands that bind at the protein-protein interface and inhibits the interaction of SARS-CoV -2 S-glycoprotein and the ACE2 receptor. This represents the first step of a translational research project to shortlist potential binders to be followed by in vitro and in vivo studies and finally clinical testing and repositioning. A further level of validation has been incorporated by considering historical evidence of biological activity of experimental ligands known to disrupt the viral host (S-RBD) and receptor (ACE-2) interaction. We have been able to show that experimental agents with biological activity also express binding affinity using our employed docking protocol.*

  

- The manuscript could benefit from a more comprehensive discussion of the limitations and assumptions of the computational methods employed. Addressing factors such as binding site flexibility, solvent effects, and conformational changes could enhance the interpretation of the results and their relevance to real-world scenarios.

*We thank the reviewer for this observation and have included the following paragraph to the discussion section:*

*The current study benefited from a cross-docking protocol that utilized two different programs with two different algorithms to validate the results of the predicted docking poses of potential binders and increase the likelihood of getting satisfying results when proceeding with future laboratory and clinical testing. This was also further supported with a MD simulation for the best hits. MD simulation addresses important factors not included in the docking calculations such as binding site flexibility, solvent effects, and conformational changes, and thereby confirms the stability and the accuracy of the predicted docking conformations and increases the probability of being adopted in a real biological environment. Of interest a number of hit ligands identified in the study have been shown in the literature to express verified experimental antiviral activity, and this study sheds light on their plausible mechanisms of action. While the in-silico methodology employed in this study is comprehensive and supports reported evidence of potential antiviral activity for some hit ligands, future studies are required to validate activity and clinical efficacy.*

  

- The study focuses on a specific set of compounds and interactions, which may limit its generalizability to other drug candidates or viral strains. Discussing the broader implications of the findings and potential applicability to related viruses or drug targets would enrich the manuscript.

*Thank you for this important observation. While drug repurposing is a well-known drug discovery strategy that gained high attention and applicability during the past few decades, this research represents a rational drug discovery project that focuses on a specific protein-protein interaction for the challenging new virus. COVID-19, in an attempt to overcome potential future outbreaks and devastating pandemic. The generalizability of this approach is highlighted; specifically, the benefits of utilizing in silico platforms towards a repurposing strategy have been highlighted in the text. It is important to note that our hit compounds were selected based on a detailed analysis of their pharmacological profiles and suitability for translation towards a clinical setting. While a number of similar studies report compounds based mainly on favorable binding scores we carefully filtered through the hit compounds and determined those with complementary pharmacological and safety profiles. It is this comprehensive approach we hope will improve the clinical translatability of our findings.*

  

- It would be beneficial for the authors to provide information on the availability of the datasets, software codes, and molecular structures used in the study. Enhancing data accessibility would promote transparency and reproducibility in computational drug discovery research.

*Thank you for this comment, we have now included the following section to the materials and methods:*

*The crystal structures analysed during the current study are available in the PDB database, [PDB ID: 6M0J]”. The molecular structures of the compound’s datasets used were obtained from DrugBank database (* *https://go.drugbank.com/drugs* *)*

*and Zinc database (* *https://zinc.docking.org/substances/subsets/fda/* *)*

  

- While the selected compounds show promise in silico, their clinical feasibility and safety profiles need to be thoroughly evaluated. Discussion of potential side effects, pharmacokinetic properties, and drug interactions would provide a more comprehensive assessment of their suitability for clinical trials.

*We thank the reviewer for this helpful comment. We have accordingly included additional data on the pharmacokinetic profiles for the top hit ligands. It is important to note that the current research utilized ligands from worldwide approved and USFDA approved drugs and nutraceuticals, all of which are clinically used medications or natural compounds with known drug-drug interactions, pharmacokinetic, and toxicity profiles. We have in the discussion highlighted the clinical translatability and significance of repurposing drugs such as Lymecycline and Hexoprenaline in the clinical setting COVID-19 and their plausible applicability. The clinical feasibility and limitations of select hit compounds are discussed in light known clinical and experimental findings. Our repurposing approach in this study aims to gives the work more translatability towards a clinical setting.*

# Reviewer report: Reviewer response for version 1

**DOI:** 10.5256/f1000research.144356.r244892

**Published:** 2024-03-01

**Authors:** Prateek Kumar

**Affiliations:** 1 University of Allahabad, Prayagraj, India

Review Report:

 The title of manuscript is interesting but it requires major revisions before getting **approved**. Authors are requested to incorporate following suggestions:

 Major revisions:

 1. Validation of docking results is missing, please include the validation of molecular docking.

 2. Perform ADMET analysis to check the drug abilities/potential of screened compounds.

 3. Perform binding free energy calculations MM/GBSA.

 Minor revisions:

 4. Improve the conclusion part in Abstract as well as the final conclusion. It should be more conclusive not broad.

 5. Manuscript require thorough checking of Grammer and typo errors.

Is the work clearly and accurately presented and does it cite the current literature?

Partly

If applicable, is the statistical analysis and its interpretation appropriate?

Not applicable

Are all the source data underlying the results available to ensure full reproducibility?

Partly

Is the study design appropriate and is the work technically sound?

Partly

Are the conclusions drawn adequately supported by the results?

Partly

Are sufficient details of methods and analysis provided to allow replication by others?

Yes

Reviewer Expertise:

Bioinformatics, Molecular Biology

I confirm that I have read this submission and believe that I have an appropriate level of expertise to confirm that it is of an acceptable scientific standard, however I have significant reservations, as outlined above.

## References

[1] : Natural products from Streptomyces spp. as potential inhibitors of the major factors (holoRdRp and nsp13) for SARS-CoV-2 replication: an in silico approach. *Arch Microbiol* .2024;206(2) : 10.1007/s00203-023-03820-5 88 10.1007/s00203-023-03820-5 38305873

# Response

**Published:** 2024-07-15

**Authors:** dania hussein

**Affiliations:** Imam Abdulrahman bin Faisal University, Saudi Arabia

*We thank the esteemed reviewer for his valued input and comments. We have addressed and responded to each comment accordingly:*

***Major revisions:***

**1.** **Validation of docking results is missing, please include the validation of molecular docking**.

*We thank the esteemed reviewer for their valuable comments and helpful suggestions. We have added a additional paragraph regarding the protocol validation. Due to the absence of any verified crystal structure of ligands bound to the SARS-Cov-2 receptor binding domain and ACE2 interface we utilized the Site Score tool on Maestro to identify and calculate the region with the highest druggability that encompasses the interactions of interest. The key interactions between the viral host and receptor have been determine and verified and we were keen to identify agents that could disrupt such interactions. Using SiteScore the binding pocket with thus determined and for validation we employed 2 different docking platforms: Glide and AutoDoc Vina. For an additional level of validation supported by experimental evidence ligands with verified activity against the S-glycoprotein-ACE2 interaction were docked using Glide. Biological activity was assessed and IC50 values determined in previous studies, this was compared to the determined binding affinities represented by the docking scores attained using the extra precision docking protocol. The findings provided another tier of validation highlighting a complementary association between the calculated binding affinities and experimental activity of our designated control ligands, this data is available in the Extended Data file.*

**2.** **Perform ADMET analysis to check the drug abilities/potential of screened compounds.**

*Thank you for this suggestion ADMET values were derived for the top seven ligands are presented as a table in the manuscript.*

**3.** **Perform binding free energy calculations MM/GBSA.**

*Thank you for this valuable suggestion, we have accordingly preformed free energy calculations and added this data to the manuscript.*

***Minor revisions:***

**4. Improve the conclusion part in Abstract as well as the final conclusion. It should be more conclusive not broad.**

*The abstract conclusion part was rewritten as follows:*

*Our study represents a rational drug discovery approach that utilizes a unique and comprehensive approach to identify agents that can bind to the S-glycoprotein-ACE2 interface using multiple computational platforms. Seven hits were identified: lymecycline, hexoprenaline,  pentagalloylglucose, polydatin, tricrocin, setmelanotide and forsythiaside. Among the hit compounds shortlisted in this study, both lymecycline and hexoprenaline were considered to be two significant drug hits in terms of their applicability in COVID-19 clinical settings and therefore are considered primary candidates for translational and clinical studies to assess their therapeutic potential in the management of COVID-19 infections*.

*The conclusion part was rewritten as follows:*

*There have been a large number of docking studies published in the literature that have identified agents that may potentially be repurposed towards inhibiting SARS-CoV-2 targets. However, our study represents a unique and comprehensive approach to repurpose drugs that can bind to the viral S-glycoprotein-ACE2 binding interface using multiple platforms. Two different docking platforms were utilized and binding free energy calculations and molecular dynamic simulation studies were performed to identify a consistent binding pattern that appears to be common in the most effective agents that have the potential to inhibit the S-glycoprotein-ACE2 interaction. Seven drugs were identified as hits including, lymecycline, hexoprenaline, pentagalloylglucose, polydatin, tricrocin, setmelanotide and forsythiaside. The hits identified in this study were additionally shortlisted for their suitability to be translated to a clinical COVID-19 setting by understanding their toxicity profile and identifying agents with verified anti-inflammatory and anti-viral capacity. In our study lymecycline, and hexoprenaline are proposed as prime candidates for further translational, preclinical and clinical investigations for the treatment of COVID-19.*

**5. Manuscript require thorough checking of Grammar and typo errors.**

 Thank you for this helpful observation, we have accordingly revised the manuscript for mistakes.
